# Contemporary Normative Values for Five Common Static Standing Tasks on Firm and Compliant Floor Surfaces in Children Two to Thirteen Years

**DOI:** 10.3390/children12010035

**Published:** 2024-12-28

**Authors:** Nancy S. Darr, Mary Rose Franjoine, Cathey Norton, Brenda L. Young

**Affiliations:** 1 School of Physical Therapy, Belmont University, 1900 Belmont Boulevard, Nashville, TN 37212, USA; nancy.darr@belmont.edu (N.S.D.); cathey.norton@belmont.edu (C.N.); 2 Scott Bieler College of Health Professions, Daemen University, 4380 Main Street, Amherst, NY 14226, USA; 3 Global and Local Sustainability, Daemen University, 4380 Main Street, Amherst, NY 14226, USA; byoung@daemen.edu

**Keywords:** children, static balance, norms, floor surface, vision

## Abstract

Background/Objectives: Static upright tasks, including standing unsupported (SU), eyes closed (SEC), feet together (SFT), tandem (TS), and single limb (SLS), are routinely examined in children and are included in many norm-referenced measures. Existing normative values for these standing tasks may not apply to contemporary children and have not been established across wide age ranges. The primary purpose of this study was to investigate developmental trajectories of and relationships between four static standing positions (SPs [SU, SFT, TS, SLS]) in children aged 2 through 13 years who are developing typically. The effects of altered sensory input, including floor surface (firm and compliant) and vision (eyes open [SU] and eyes closed [SEC]), as well as influences of sex, height, weight, and BMI on static standing were also examined. Methods: Children (*n* = 807) developing typically performed two trials of each task up to 2 minutes per trial using standardized procedures. A total of 482 children were also tested on a compliant surface. Results: Descriptive statistics were calculated by age, height, weight, task, and floor surface. Two-way ANOVA showed no significant effects (*p* > 0.05) of sex on standing times; however, age was significant for all standing tasks. Repeated-measure ANOVA and Tukey post hoc tests identified significant effects (*p* < 0.05) of age and floor surface on standing times. SU, SEC, and SFT times increased up to 8 years, with most children achieving the 2 minute maximum by age 8. TS and SLS times improved up through 13 years, with wide variations in performance noted in children 8 years and older. Conclusions: Contemporary normative performance values are provided for five common standing tasks on firm and compliant surfaces by age in one-year increments.

## 1. Introduction

It is commonly accepted that balance is critical to everyday function. Throughout a day, a child will assume and maintain many different standing positions during functional activities. For example, when donning footwear or pants, a child may choose to stand on one foot depending on their age and environmental context. In addition, many athletic activities require a narrow base of support, e.g., a gymnast often stands on a balance beam in tandem or on one foot. These examples describe one aspect of balance, specifically static, also known as steady-state balance. A commonly accepted definition of static balance is the ability to control the body’s center of mass (COM) relative to an unchanging base of support under predictable environmental conditions [1]. Static balance tasks emphasize stability over mobility and allow for varying orientations of the body and body segments. Although one can also examine the development of dynamic balance during movement, reactive balance to perturbations, and anticipatory control, the scope of this paper will be limited to the development of static balance abilities in children.

Function demands that a child sustain standing under a variety of environmental and sensory conditions. Static positions commonly assumed by children during daily activities may include standing unsupported with feet in a variety of positions, including but not limited to shoulder width, feet together, tandem, and single-limb standing. The ability to attain and maintain these static standing positions develops throughout childhood [2,3,4,5,6]. Therapists and educators routinely examine the development of static standing balance abilities in preschoolers and school-aged children. The specific methods of measurement, including position of the child, complexity of tasks, and duration of measurement, vary across valid standardized developmental and balance assessments. Clinicians, educators, and researchers need to be aware of the variables that limit our understanding of the development of static standing balance in children.

Static standing balance in children has been assessed using a wide variety of tests and measures that range from single-item measures [3,7,8] to complex static standing items with multifaceted conditions that are frequently embedded in standardized assessments of motor development [9,10,11]. In a scoping review, Sibley, et al. examined components of standing “postural control” in a variety of pediatric balance measures and determined that 72% of pediatric balance measures had elements of static stability [12]. Instrumented measures of static standing have also been used in research and to a lesser degree in clinical practice [2,3,13,14]. Recently, technological advances have enabled some instrumentation to become portable and affordable for clinical use [15]. Wearable technology to assess balance in children is also emerging [16].

Children’s static standing ability has also been assessed within functional balance measures, which are frequently used in clinical and community-based settings. These measures may include a single construct of balance such as the stork standing test [7] or multiple constructs [17,18,19]. Variations within these measures exist relative to the specific static standing items examined and how each is administered, scored, and interpreted.

In their scoping review, Sibley et al. highlighted numerous differences in methodologies across balance assessments in children, concluding that comparative analyses or clinical interpretation of findings across studies is challenging [12]. When comparing static standing tasks across measurement tools, vast differences exist in complexity of instructions for the child, environmental setup, including equipment, as well as item administration and scoring. Parameters also vary greatly with regard to arm position [4,8,19,20], direction of visual gaze [4,19], floor surface, and body position [7,19,20]. In addition, age ranges and groupings have varied greatly across studies, making comparisons problematic [2,4,5,7,19].

The role of sensory systems in standing balance has been explored extensively in the literature. During daily activities, children must be able to stand on a variety of surfaces that provide differing amounts of stability and somatosensory input. Children also must be able to perform static standing activities in darkness or semi-darkness. Many investigators have used the Clinical Test of Sensory Interaction and Balance (CTSIB) and variations developed for use in children (p-CTSIB and modified p-CTSIB) to examine the influences of floor surfaces and vision availability on static standing balance [5,21,22,23]. It is commonly accepted that the degree of floor surface compliance and availability of ambient light influence static standing stability [5,21,23].

Time parameters for measurement of static standing vary across studies, with no clear consensus on upper boundary determination. Thirty seconds appears to be the most common maximum duration when measuring static standing across both instrumented and clinical assessments [13,17,19,22], with as little as 10 seconds used in some measures [10,19,24]. Few investigators have examined static standing beyond 30 seconds [5,7,25]. There is no consensus in the literature regarding how long a child can be expected to stand in different positions based on their age, floor surface, or lighting conditions.

Investigators have also examined the influences of height, weight, and BMI on static standing; however, height and weight are related to age, making interpretation of their relative importance difficult. There is conflicting information regarding the effect of BMI on children’s balance. Some investigators have found that children with BMI in the normal range demonstrate better balance and postural control than those at the extremes of BMI [20,24]. In a platform posturography study, Kolic et al. examined the impacts of age, height, BMI, and sex on multiple aspects of static standing balance in children aged 4 through 12 years [24]. Children’s performance improved with age on all tasks; however, effects of BMI and sex varied by age and task. Children with lower BMI outperformed those with elevated BMI, and girls tended to demonstrate more mature balance strategies at most ages. Other authors have investigated effects of sex on development of balance in children, with similarly mixed results. In 2010, Franjoine et al. examined impacts of sex on functional balance in children aged 2 through 13 years, and found that girls outperformed boys at most ages [19]. Lee and Lin found that girls aged 3 to 9, demonstrated more stability in SLS compared to boys [20], while Voss found that sex-based differences in postural sway measures varied by age [13]. Two recent systematic reviews examined age- and sex-based differences in balance in preschoolers [26] and in children aged 6 to 18 years [27]. Results of both systematic reviews suggested that girls tend to outperform boys on static standing balance tasks; however, results were inconsistent and varied by age and task [26,27].

### Purpose

A clear understanding of typical performance across a variety of static standing positions is critical for identifying children who may have challenges in static balance limiting their ability to fully participate in daily tasks. The primary purpose of this study was to investigate developmental trajectories of and relationships between four static standing positions (standing unsupported [SU], standing, feet together [SFT], tandem standing [TS], single-limb standing [SLS]) in children aged 2 through 13 years who are developing typically. The effects of altered sensory input, including floor surface (firm [FFS] and compliant [CFS]) and vision (eyes open and eyes closed), as well as influences of sex, height, weight, and BMI on static standing were also examined.

## 2. Materials and Methods

This study was approved by the institutional review boards of Belmont University (Nashville, TN, USA) and Daemen University (Amherst, NY, USA).

### 2.1. Study Design

This was a descriptive, cross-sectional study using a large cohort of children who were identified as typically developing (TD) by a parent or guardian at the time of enrollment into the study.

### 2.2. Participants and Testing Locations

The 807 children (boys = 413, girls = 394) who participated in this study should be considered a sample of convenience. They were recruited from schools, after-school programs, preschools, day care centers, and other community-based programs and resided in diverse regions of the United States, including the Midwest, Midsouth, Western New York, and the Eastern Great Lakes. Testing environments varied across communities and included classrooms, clinics, gymnasiums, hallways, homes, libraries, playgrounds, religious facilities, schools, and other community sites.

### 2.3. Inclusion and Exclusion Criteria

Children aged 2 through 13 years were eligible to participate in this study if they could stand without external support for 4 seconds and follow simple, one-step instructions. Children were excluded from participation if they experienced any of the following: a communicable illness within the previous 2 weeks, a medical condition that resulted in a physician-recommended activity restriction, an orthopedic injury within the prior 3 months, a seizure within the previous 3 months, or other health conditions resulting in developmental delays or disabilities as indicated by parent on the health history form (HHF).

### 2.4. Raters and Rater Training Process

Data collection was conducted by 4 physical therapy (PT) faculty mentors, and 64 entry-level Doctor of Physical Therapy students (total raters = 68). Prior to engaging in data collection, all student raters participated in a standardized training program and achieved a mastery level of skill that included both cognitive and psychomotor assessment of their learning. The training and assessment process was conducted by PT faculty mentors, who were experienced clinicians and educators. It emphasized the setup of each testing condition, safety of the child, and accuracy of timing of static balance for each standing task on a firm floor surface (FFS) and compliant floor surface (CFS). Interrater reliability for the 68 raters determined by interclass correlation coefficients was excellent (ICC = 0.946–0.997). All student raters were directly supervised by faculty during data collection.

### 2.5. Instrumentation

To ensure the consistency of floor surfaces during testing across multiple community sites, a standardized testing surface was created. This standardized setup included the use of a 45.72 × 60.96 cm (18 × 24-inch) entryway rug with two, 2-inch-wide taped lines and a 40 × 48× 6 cm (20 × 16.4 × 2.5-inch) Airex™ foam balance pad. The taped lines were affixed perpendicularly to one another on the rug, extending the full length and width of the rug to form a “+” sign. For testing on the FFS, the rug with markings was used on the existing floor surface of the community site. To create the CFS, the rug was placed over the foam balance pad on the community site’s existing floor surface. For older children (adolescents) with long feet, two foam pads were used to create a surface that accommodated their foot size.

### 2.6. Procedure

Prior to data collection, each child’s parent/guardian completed an informed consent, which included permission to video-record the testing session, as well as an HHF. Assent to participate was obtained from children 7 years and older using an age-appropriate pediatric assent form. A waiver of assent was obtained from the parent/guardian for children 6 years and younger. The HHF was used to identify children who should be excluded from participation and determined if they met the medical and/or developmental criteria for exclusion. To ensure all children met the minimum inclusion criteria, each child was asked to stand without external support for 4 seconds, and caregivers were asked if the child could follow 1-step directions. The child’s date of birth was also verified with the caregiver.

Prior to examination of the child’s static balance abilities, their height and weight were measured and recorded (Table 1). Each child was tested individually and was asked to stand in four different standing positions (SPs) on the FFS. The SP included: standing unsupported with feet shoulder-width apart (SU), standing with feet together (SFT), tandem standing (TS) and single-limb standing (SLS). Additionally, the influence of vision on unsupported standing with eyes closed was examined (SEC). A subsample of the 807 children (*n* = 482) also performed the same 5 standing activities on the CFS (Table 1). Participant demographic values (height, weight) are also presented using the United States customary system of measurement (Appendix A). All testing was video recorded.

A description of the standing tasks and the standardized child instructions for each standing task is provided in Table 2. Raters were encouraged to adapt the instructions to meet the child’s age, language skills, and developmental abilities. For each standing task, the child received individualized verbal instruction and a visual demonstration. A practice trial for each standing task was provided to ensure the child understood the expectations of the task. The rater was able to reinstruct the child, if needed, during the practice trial for that task. Additional feedback, other than general positive reinforcement, was not provided during testing. The rater stood close to the child and actively guarded to ensure the child’s safety during the practice and testing trials.

Each child was expected to independently assume each standing task as described in Table 2, stand with feet still in the appropriate test position, and not touch anything with their hands during the trial. The child did not receive specific instructions for hand placement or direction of eye gaze. Children were asked to stand in each position for 2 minutes (120 seconds). They were allowed up to 2 trials to achieve 120 seconds. If the child achieved 120 seconds on the first trial, the 2nd trial was omitted. The rater was able to engage the child in simple conversation to sustain their performance during the task. A trial was terminated if the child used any part of their body for support other than the weight-bearing feet (foot), if the examiner touched the child to prevent a fall, or if there was movement of any part of the weight-bearing feet (foot) in space off the support surface. Table 2 provides additional task-specific criteria for the termination of trials. Balance responses within the feet (foot) or subtle weight shifts in feet (foot) or toes were acceptable and did not cause trials to be terminated. Times were recorded to the one-hundredth of a second.

## 3. Results

**Figure 1:**
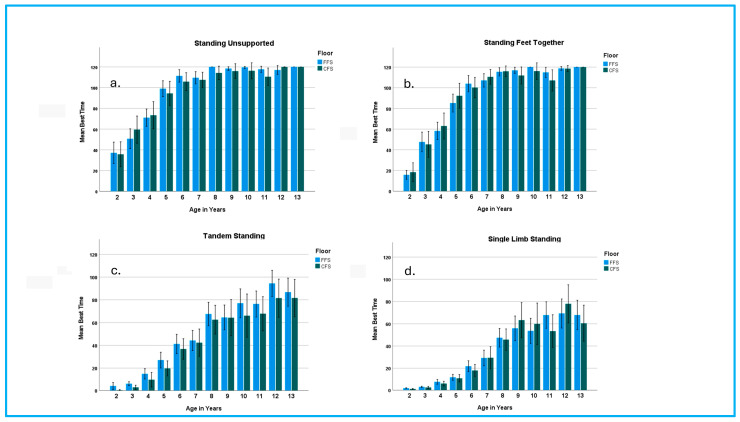
Effect of Standing Position, Age, and Floor Surface on Standing Times. This figure provides means (±95% confidence interval) for best times in seconds for each standing position by age group (in years) and floor surface. Positions are: (**a**) Standing Unsupported, (**b**) Standing Feet Together, (**c**) Tandem Standing, and (**d**) Single-limb Standing.

Children were divided into 12 groups based on one-year age increments for all data analyses. Descriptive statistics, utilizing the best of two trials, were calculated by age group for each standing position on both the FFS and CFS (Table 3), as well as for height, weight, and BMI (Table 1, Appendix A). Children’s performance improved in all SPs on both the FFS and CFS between ages 2 and 13 years. The degree of change varied with SP, floor surface, and age (Figure 1). With the exception of a few children in the younger age groups, all children were able to perform SU and SFT on both the FFS and CFS. However, children in the youngest age groups were challenged by TS and SLS on both surfaces (Table 3). Less than 50 percent of children in the 2- and 3-year-old groups were able to perform either position on the CFS.

For each SP, a two-way ANOVA with Tukey HSD post hoc analyses was used to examine age and sex effects on standing times. The results indicated a significant age effect (*p* < 0.001). The effect of sex was not significant at most ages (*p* > 0.05) (Appendix B), with the exception of TS times on the FFS in girls, which were significantly greater than boys across age groups (*p* = 0.029). SU and SFT times increased between ages 2 and 8 years, with most children achieving the 2-minute maximum by age 8 (Figure 1). TS and SLS times improved through 13 years, with wide variations in performance noted in children 8 years and older. These results are based on the total number of children willing to attempt each task at each age (Table 3).

**Figure 2:**
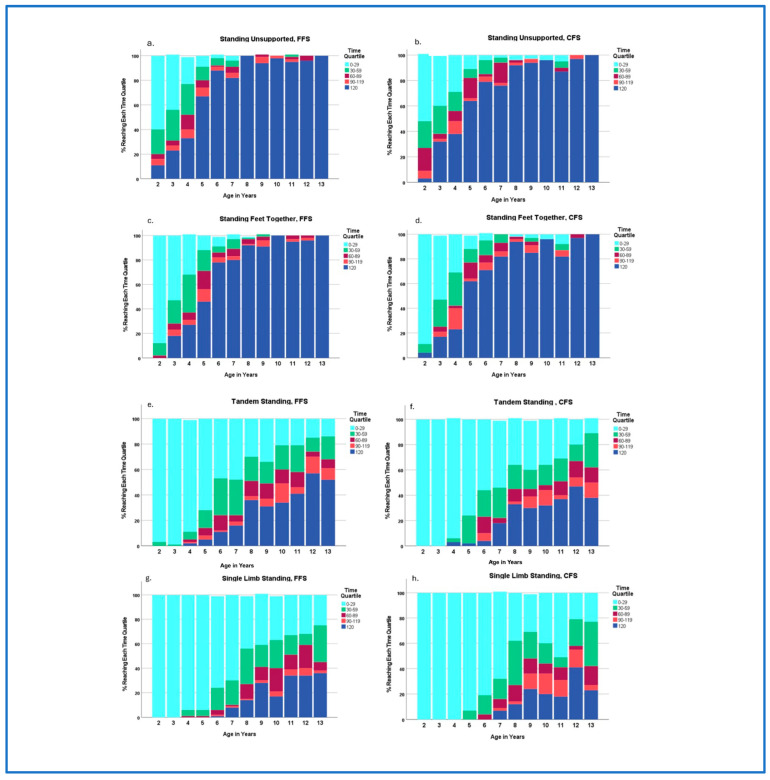
Percentage of Children in Time Quartiles for each SP and Floor Surface by Age Group. Quartiles represent intervals of standing times in seconds (0–29, 30–59, 60–89, 90–119) and those attaining the maximum time of 120 seconds, as these children could potentially have maintained the position longer. (**a**) Standing Unsupported (SU) on Firm Floor Surface (FFS), (**b**) SU on Compliant Floor Surface (CFS), (**c**) Standing Feet Together (SFT) on FFS, (**d**) SFT on CFS, (**e**) Tandem Standing (TS) on FFS, (**f**) TS on CFS, (**g**) Single-limb Standing (SLS) on FFS, and (**h**) SLS on CFS.

Children’s performance on each SP is also presented by time quartiles (Figure 2). As some children reached the maximum time of 120 seconds for various SPs, quartile groups were determined to represent percentages of children achieving up to 25% of the time (30 seconds), 50% of the time (60 seconds), 75% of the time (90 seconds), and 99% of the time (119 seconds), and those achieving the maximum time for each age group. This representation allowed us to consider the age-related progression in achieving each task and the relative difficulty of each position across age groups. More than 60% of children achieved the maximum score of 120 seconds by age 5 for SU on both floor surfaces and by age 6 for SFT. In contrast, less than 60% of children were able to achieve the maximum time for TS or SLS on either surface across all ages (Figure 2). After age 6 years, standing times were not significantly different between age groups for SU and SFT (Appendix B). Trajectories of skill acquisition for TS and SLS differed from each other and from the other SPs (Figure 2).

Pearson correlation coefficients showed limited relationships between SPs within each age group and floor surface: the majority of correlation coefficients were poor to fair for a given floor surface for a given age (for example age 5 *r* = 0.176 to 0.610). The strongest correlations were observed between SU and SFT (*r* = 0.493 at age 2 and 0.743 at age 6) on the FFS. TS and SLS demonstrated moderately strong correlations at age 4 (*r* = 0.531) and age 7 (*r* = 0.605) on the FFS.

Two-way repeated measure ANOVAs with Tukey HSD post hoc analyses were used to identify significant effects of age and floor surface on standing times. For all SPs, floor surface and age effects were significant (*p* < 0.001) (Appendix C). Across all ages and SPs, standing times were significantly longer on the FFS than CFS; however, for SLS, there was a significant interaction between floor surface and age (*p* = 0.006).

**Figure 3:**
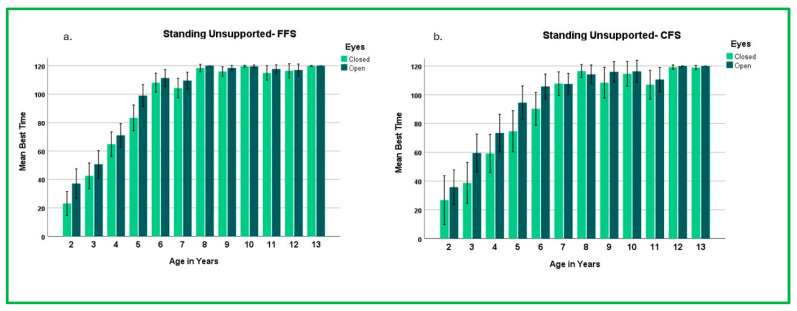
Effect of Altered Sensory Input and Age on Standing Time. Mean standing times (±95% confidence intervals) for standing unsupported (SU) with eyes open and closed by age group on: (**a**) Firm Floor Surface (FFS) and (**b**) Compliant Floor Surface (CFS).

Times were significantly greater when standing with eyes open than with eyes closed on both the FFS and CFS (*p* < 0.001), as indicated by two-way repeated-measure ANOVAs with Tukey HSD post hoc analyses (Appendix D). The ability to use vision to assist with balance (eyes open vs. eyes closed) affected performance differently across age groups depending on the floor surface (interaction *p* = 0.015 to <0.001, Figure 3). Young children were challenged by SEC on both surfaces, with the CFS being more difficult than the FFS. Even in the oldest age groups, some children were not able to perform SEC on either surface for the maximum time (Appendix E). However, 70% of children were able to stand for at least 30 seconds on the FFS by age 4 and on the CFS by age 5 years (Appendix F).

To investigate contributions of height, weight, age, and BMI on performance for each SP, a backward multiple linear regression was performed for each floor surface. Due to the collinearity between height and weight, weight was removed as a variable in the models. These regressions indicated that across all SPs and each floor surface, age was the only variable with a consistent significant effect (*p* < 0.001). The model R^2^ values were low (0.294–0.437). Predictive models and the significance of each independent variable for each SP and floor surface are provided in Appendix G.

## 4. Discussion

To the best of our knowledge, this is the first study to report children’s ability to maintain multiple standing positions (SU, SFT, TS, SLS) across a wide age range (12 years) in one-year increments in a large sample. Children’s performance was assessed for up to 2 minutes per position on firm (FFS) and compliant (CFS) floor surfaces. Previous studies have examined smaller samples of children, combined age groups, and/or examined a smaller repertoire of static standing tasks [2,4,5,7,8,17].

With intention, the methodology of this study was designed to place the emphasis on static standing balance and to minimize other confounding variables that may detract from children’s ability to maintain focus on their balance. Each child was tested individually in a relatively distraction-free environment. Directions were intentionally designed to be simple and easily understood (Table 2). Language and cognitive abilities were also considered when instructing the child, and care was taken to individualize instructions as needed. Examiners provided demonstrations of each task, and a practice trial was utilized to ensure the child understood task expectations. When developing each standing task, we intentionally limited instructions to the essential aspects for each SP and minimized extraneous task requirements. Specific instructions for eye gaze and arm position were not provided to minimize cognitive load, enabling the child to maintain maximum focus on standing. As noted in Table 2, the child was able to self-select their weight-bearing foot for SLS and lead foot for TS. For similar reasons, exact specifications for positioning of the non-weight-bearing leg in SLS were not provided other than not to touch legs together. Our methodology contrasts with that of many other studies in which visual gaze, arm position, and other positional factors were more constrained and potentially drew focus away from maintaining the static standing position(s) [4,5,8,20,22].

Many investigators have placed children into age groups of two- [5], three- [4], or more year increments [2] for the purpose of examining static standing abilities. We chose to group children into one-year increments to enable clinicians and educators to easily utilize the results of this study. This decision was supported by our large sample and preliminary data analyses. We acknowledge that stratifying children into artificial one-year increments may cause challenges in applying the results of this study to individual children at the very upper and lower ends of each age band. Children who are relatively close in age and ability may be in different age groups. For example, a 35-month-old child’s standing abilities would be compared with other 2-year-olds, while a child who is chronologically one month older would be considered a 3-year-old.

Most investigators have examined standing times in children for 30 seconds or less, with very few reporting times beyond 30 seconds. Latorre Román et al [7] examined SLS for up to 60 seconds in preschoolers using the stork standing test. Each child was required to stand on one leg with their non-weight-bearing foot in contact with the inside of the weight-bearing leg. We used a more traditional SLS position, as described in Table 2. Condon and Cremin examined SLS and TS on an FFS and a 6-inch foam surface in children aged 3 to 13 years for up to 120 seconds [5]. They grouped children in 2-year age increments for analyses and provided limited description of their specific methodology. The methodological designs of these studies make direct comparisons to our findings challenging.

Many of the existing standing balance measures use maximum times of 10 to 30 seconds, which may fail to capture true performance capabilities or identify challenges for children as they age [13,17,24]. Children aged 3 years and older were easily able to perform SU and SFT for more than 30 seconds. Children’s performance on these relatively easy tasks markedly increased through age 8 with continued nonlinear gains through at least 13 years of age (Figure 1). TS and SLS are more difficult for children than SU and SFT; however, mean standing times for TS and SLS exceeded 40 seconds for 6- and 8-year-olds, respectively. Mean standing times gradually increased for each of these tasks through age 12 years (Table 3), although the pattern and trajectory of improvement was different for each task (Figure 2). The results of this study support the potential benefits of measuring static standing times beyond the traditional 30 seconds. A 2 minute time frame was chosen in this study for each item to capture the potential range of performance. Many children in the older age groups reached and easily could have exceeded the 2 minute time parameter. A question that should be asked is: How long is long enough to examine static standing tasks in children? The answers to this question may be influenced by the child’s age, their health status, the setting, and the goals of the assessment.

Age was the best predictor of children’s performance for each SP (Appendix C and Appendix G); however, time trajectories were nonlinear within each SP and uniquely differed between the four SPs. As seen in Figure 1, SU and SFT improvements in standing times were greatest between ages 2 and 8, with more gradual change up through age 13 years. Quartile graphs highlight the nuanced differences in performance between SU and SFT on both floor surfaces (Figure 2). For example, 65% of 5-year-olds were able to perform SU for 120 seconds, whereas only 46% of this age group were able to perform SFT for the maximum time.

TS and SLS are both challenging tasks for children with very different performance trajectories. Mean TS and SLS times first surpassed 10 seconds at ages 4 and 5, respectively, and improved up through at least age 12 years (Figure 1). Quartile analyses (Figure 2) reveal different trajectories of skill acquisition for TS and SLS on both the FFS and CFS. When examining the percentage of children who can stand for at least 30 seconds (second quartile), SLS improvements were more gradual and stepwise in one-year increments, whereas TS improvements appeared to occur in two-year increments (Figure 1). Based on the performance quartiles (Figure 2) and lack of strong correlations between standing times for age, floor surface, and SP, there were limited relationships between standing positions. These findings support examination of all four SPs to obtain a comprehensive static balance assessment, as trajectories within each SP were unique and relationships between SPs were limited. The results support that performance on one SP cannot be used to predict performance on other SPs. It may also be important to consider examining other SPs based on the individual’s goals and the setting.

Many environmental factors may affect static standing balance, including floor surface and ambient light conditions. Children performed significantly better on the FFS than on the CFS in all four SPs (Table 3). These findings are consistent with other investigators who have found reduced standing times on the CFS, especially pronounced when eyes are closed [5,21,22,23]. Interaction between age and floor surface was significant for SLS, most likely due to marked variability in performance by age and floor surface in children 9 years and older. This appeared to be the most difficult SP for all children, which may account for much of the variability. Based on these results, the authors recommend taking care to ensure consistency of floor surface compliance when assessing standing times in children. In addition, examination of standing on multiple floor surfaces may be important in a comprehensive assessment of children’s static standing balance.

Consistent with previous studies, children were able to stand significantly longer when vision was available to them (SU) than when they were blindfolded (SEC) on both the FFS and CFS. This was especially pronounced in the younger age groups. Performance differences in eyes-open and -closed conditions were significant between age groups on the FFS in one-year age increments through 5 years and in two-year age increments through age 7 on the CFS (Figure 3, Appendix E).

Standing times across all ages on the FFS were longer with eyes open; however, on the CFS at ages 7 and 8, standing times were longer with eyes closed. This variation at ages 7 and 8 years may be due to changes in how children manage sensory information [26]. Although performance varied, by age 3 years, mean standing times were greater than 30 seconds with eyes open and eyes closed on the FFS and CFS (Figure 3, Appendix E and Appendix F). Historically, a 30-second maximum standing time has been used when examining the relative contributions of vision (eyes open vs. eyes closed) and somatosensory information (FFS vs. CFS) on static standing balance. This metric may be insufficient to identify children who have difficulty utilizing sensory information to modulate their ability to stand. Therefore, it may be necessary to either increase the standing times beyond 30 seconds or utilize more challenging SPs to identify children who have difficulty managing sensory input to maintain static standing balance [22,23].

Other investigators have demonstrated variable effects of height, weight, and BMI on static standing balance in children. Interpretation of these studies can be challenging because height and weight increase with age and BMI varies developmentally. The medians across all age groups for boys were near the 50th percentile provided by CDC data [28]. Girls demonstrated more variability in BMI with 2, 3, 12, and 13-year-olds exceeding the 75th percentile. The remaining age groups had medians close to the CDC 50th percentile.

The majority of participants in this study were within the normal range for BMI, with only 4.6% of boys and 2.5% of girls overweight and less than 1% of either sex underweight. Therefore, we cannot make any comparisons of performance based on BMI within this study. The anthropometric data derived from our study provide evidence that subjects were within a healthy range, and thus no negative or positive impact on balance would be anticipated.

Differences in static standing times between boys and girls were not significantly different across age groups, SPs, or floor surfaces, with the exception of TS on the FFS, where girls outperformed boys at most ages. This result contrasts with our previous study, where we found that girls outperformed boys on the Pediatric Balance Scale (PBS) in most age groups (2 through 13 years) [19]. The PBS contains 14 items and yields a composite balance score. It should be noted that the maximum standing times on the PBS for the static standing tasks are 30 seconds for SU, SFT, and TS and 10 seconds for SLS and SEC. Most studies that have examined differences in standing balance between boys and girls have utilized time frames ranging from 10 to 30 seconds [20,26,27].

Even for highly skilled investigators, it was difficult to maintain the attention of a child for 2 minute in static standing for up to two trials of each of the five tasks under two different floor conditions. Raters were trained to engage the children in non-stressful conversation to help pass the time. At times, children had to be redirected to avoid becoming overly animated in their conversation causing them to become unstable. Young children, especially the 2-year-olds, were difficult to test and would frequently walk away when they became bored or frustrated with the task. Older children in the 11- to 13-year-old groups demonstrated boredom and frustration differently and may not have always performed to their true potential. In addition, samples were relatively smaller in the older age groups, which can lead to a more robust impact of children not interested in standing the entire two minutes (Table 1, Appendix A). In examining the HHF information provided by caregivers, there were no criteria present for removing any of the children who exhibited questionable behavior during testing.

Except in the youngest age groups, it was very rare for children to refuse to attempt any of the standing tasks on either the FFS or CFS (Table 3, Appendix E). Children as young as 2 were willing to perform SU and SFT on both surfaces. Although children in the 2- and 3-year-old groups were often unable to perform SLS or TS on the CFS, they were willing to attempt these tasks on both surfaces. However, when children were blindfolded in the SEC tasks, many were reluctant to participate. Young children were frequently unwilling to keep the blindfold in place and would remove it. Older children found creative methods to peak around the blindfold. Timing was always stopped when either the child removed the blindfold or was in any other way able to use their vision. Raters were trained to apply the blindfolds quickly and provide reassurance to reduce frustration, as well as to be aware of the many strategies children used to gain access to visual cues.

## 5. Conclusions

The primary purpose of this study was to investigate developmental trajectories of and relationships between four SPs (standing unsupported [SU], standing feet together [SFT], tandem standing [TS], and single-limb standing [SLS]) in children aged 2 through 13 years who are developing typically. This study provides clinicians and educators normative values (means, standard deviations, and 95% confidence intervals of the means) for five static standing tasks on the FFS and CFS. These values will be useful to clinicians and educators when comparing a child’s static standing abilities to their age-matched peers. To obtain a comprehensive assessment of a child’s static standing abilities, the authors recommend testing five standing tasks (SU, SFT, TS, SLS, and SEC) for at least 120 seconds.

## Figures and Tables

**Table 1:** Demographics of study participants.

Age	N		Height	Weight	BMI	Floor Surface (N)	
Years	Boys	Girls	Mean (SD)	Mean (SD)	Mean (SD)	FFS	CFS
2	30	26	89.8 (6.40)	14.2 (2.35)	17.8 (3.42)	56	34
3	41	39	98.5 (6.46)	16.6 (3.14)	17.1 (2.39)	80	50
4	42	57	105.8 (6.22)	18.5 (3.08)	16.4 (1.91)	99	48
5	40	41	113.3 (6.41)	21.0 (4.10)	16.3 (2.41)	81	45
6	31	42	120.5 (7.74)	23.6 (4.58)	16.1 (2.19)	73	53
7	40	39	126.7 (7.28)	26.9 (4.62)	16.8 (2.47)	79	45
8	45	33	132.7 (8.49)	30.9 (7.18)	17.5 (3.19)	78	53
9	31	34	136.6 (10.16)	34.2 (8.15)	18.2 (3.47)	65	33
10	27	20	144.2 (6.55)	37.0 (8.26)	17.7 (3.02)	47	25
11	36	22	150.0 (12.48)	41.4 (10.58)	18.3 (3.63)	58	39
12	28	19	157.8 (9.62)	50.4 (10.96)	20.1 (3.49)	47	31
13	22	22	161.1 (13.64)	54.4 (14.57)	20.8 (4.03)	44	26
**Total**	**413**	**394**				**807**	**482**

Table 1 provides a description of study participants including their distribution by age in one-year increments, and includes means (standard deviations) for height, weight and BMI per age group. Height is reported in cm and weight in kg. BMI was calculated using the following formula: BMI = weight (kg)/height (cm) squared. Additionally, the number of participants is provided for each floor surface (firm floor surface [FFS] and compliant floor surface [CFS]) by age group.

**Table 2:** Descriptions of standing tasks, child instructions and additional criteria for trial termination.

Description of Standing Activity	Child Instructions	Additional Criteria for Termination of the Trial
**Standing Unsupported:** Stand with feet shoulder width apart on the line	“*Stand very still with your toes on this line. Do not move your feet*.”	No additional criteria
**Standing, Feet Together:** Stand unsupported with feet together on the line	“*Stand very still with your feet together and your toes on this line. Do not move your feet*.”	Movement of either foot so that the inside borders are no longer completely in contact with each other
**Tandem Standing:** Stand with one foot in front of the other, in a heel-to-toe position on the line. The child self-selects which foot to place in front. The heel of the front foot should be in contact with the toes of the rear foot. Both feet should be placed on the line, keeping feet in the sagittal plane. When standing on the CFS, a second foam pad may be necessary for children with longer feet.	“*Put one foot on the line. Now put your other foot on the line with your heel touching the front of your toes. Keep your feet very still*.”	Movement of either foot out of the tandem position, including a slight turn of either foot inward or outward
**Single-Limb Standing:** Stand with one foot placed on the line. The child self-selects which foot to stand on and the position of the non-weight-bearing limb.	“*Stand on one foot on this line. Try not to move the foot you are standing on.* *Do not let your legs touch.”*	Movement of the non-weight-bearing limb creating contact with the weight-bearing leg
**Standing with Eyes Closed:** Stand unsupported with feet shoulder-width apart on the line. A single-use blindfold is placed over the eyes and secured. Verify that the blindfold is secure and the child cannot see below, above, or out the sides of the blindfold.	“*When I say close your eyes, I want you to stand still, close your eyes, and keep them closed until I say open your eyes.”*	Movement of the head, face, or eyebrows that displaces the blindfold, or removal of the blindfold by the child.

Table 2 provides a description of the standing tasks including guidelines for administration, child-centered instructions and specific criteria for termination of the timed-trial for each task.

**Table 3:** Standing times by age and floor surface for standing unsupported, standing with feet together, tandem standing, and single-limb standing.

		Standing Unsupported Time					Standing, Feet Together Time				
Age	Surface	% Able to do Task	Mean (SD)	Range	95% CI		% Able to do Task	Mean (SD)	Range	95% CI	
					Lower	Upper				Lower	Upper
					Bound					Bound	
2	FFS	98.2	37.1 (38.44)	117.9	26.68	47.46	92.7	15.7 (15.18)	62.5	11.47	20.01
	CFS	100	35.7 (34.39)	119.0	23.73	47.73	82.4	18.3 (23.57)	120.0	9.19	27.47
3	FFS	100	50.7 (43.24)	117.4	41.05	60.30	97.5	47.6 (42.26)	120.0	38.05	57.11
	CFS	100	59.5 (46.16)	118.5	46.38	72.62	92	45.2 (42.32)	120.0	32.65	57.79
4	FFS	100	71.0 (41.91)	117.4	62.66	79.38	99	58.3 (41.90)	117.6	49.87	66.67
	CFS	100	73.4 (44.94)	115.0	60.36	86.46	100	63.0 (43.32)	118.5	50.43	75.58
5	FFS	100	99.0 (34.76)	104.5	91.30	106.67	100	85.2 (39.38)	117.2	76.49	93.91
	CFS	100	94.5 (38.72)	113.5	82.85	106.12	100	92.3 (40.19)	116.6	80.18	104.33
6	FFS	98.6	111.3 (25.49)	114.0	105.33	117.31	98.6	103.9 (33.87)	120.0	96.03	111.84
	CFS	100	105.8 (31.20)	104.8	97.20	114.40	98.1	100.3 (35.24)	120.0	90.44	110.06
7	FFS	100	109.5 (26.29)	98.7	103.64	115.41	100	107.1 (28.85)	103.0	100.65	113.57
	CFS	100	107.5 (24.64)	98.8	100.09	114.90	100	110.6 (23.75)	89.2	103.45	117.72
8	FFS	100	120.0 (0.00)	0.0	120.00	120.00	98.7	115.4 (17.43)	104.8	111.46	119.38
	CFS	100	114.2 (23.61)	119.0	107.70	120.71	98.1	116.0 (18.56)	117.0	110.78	121.11
9	FFS	100	118.4 (7.13)	45.0	116.66	120.19	100	116.9 (12.18)	76.3	113.84	119.88
	CFS	100	115.9 (20.19)	115.0	108.78	123.10	100	111.9 (23.29)	99.5	103.64	120.15
10	FFS	100	119.5 (3.21)	22.0	118.59	120.47	100	120.0 (0.00)	0.0	120.00	120.00
	CFS	100	116.3 (18.66)	93.3	108.57	123.97	100	116.1 (19.44)	97.2	108.09	124.14
11	FFS	100	117.7 (11.39)	67.5	114.70	120.69	100	114.9 (18.85)	96.8	109.95	119.86
	CFS	100	110.6 (25.90)	95.0	102.16	118.95	100	107.1 (31.78)	113.0	96.78	117.38
12	FFS	100	117.0 (14.41)	75.3	112.77	121.23	100	118.7 (6.27)	36.8	116.89	120.57
	CFS	100	119.9 (0.34)	1.9	119.81	120.06	100	118.5 (8.26)	46.0	115.49	121.55
13	FFS	100	120.0 (0.00)	0.0	120.00	120.00	100	120.0 (0.00)	0.0	120.00	120.00
	CFS	100	120.0 (0.00)	0.0	120.00	120.00	100	120.0 (0.00)	0.0	120.00	120.00
2	FFS	70.9	4.0 (10.07)	57.8	0.73	7.26	83.6	1.7 (1.61)	6.0	1.22	2.18
	CFS	26.5	0.3 (0.87)	2.6	−0.38	0.96	44.1	1.0 (0.92)	2.6	0.50	1.52
3	FFS	87.5	6.1 (7.30)	32.0	4.33	7.81	91.3	2.9 (2.74)	15.0	2.30	3.58
	CFS	36	2.9 (3.82)	15.0	0.95	4.75	56	2.4 (2.62)	12.3	1.42	3.46
4	FFS	96	14.9 (22.45)	120.0	10.29	19.43	93.9	7.6 (10.68)	66.8	5.35	9.75
	CFS	81.3	9.6 (20.41)	120.0	3.02	16.25	89.6	6.0 (6.94)	28.4	3.81	8.08
5	FFS	97.5	27.0 (31.07)	120.0	20.07	33.99	98.8	11.7 (12.16)	69.1	8.95	14.36
	CFS	91.1	19.7 (21.21)	120.0	12.96	26.35	95.6	10.8 (10.96)	53.0	7.44	14.18
6	FFS	100	41.2 (36.60)	120.0	32.69	49.77	100	21.8 (21.19)	120.0	16.82	26.71
	CFS	98.1	36.8 (32.58)	119.8	27.74	45.88	100	17.9 (19.50)	83.7	12.56	23.31
7	FFS	100	44.3 (39.96)	117.7	35.32	53.22	100	29.2 (31.44)	118.3	22.18	36.26
	CFS	100	42.4 (40.12)	120.0	30.33	54.44	100	29.4 (33.28)	118.3	19.42	39.42
8	FFS	98.7	67.5 (45.31)	115.0	57.23	77.80	97.4	47.4 (36.80)	117.3	39.03	55.85
	CFS	98.1	62.6 (45.32)	114.4	49.97	75.21	98.1	45.8 (34.70)	116.3	36.08	55.41
9	FFS	100	64.6 (43.86)	113.1	53.69	75.43	100	55.9 (44.83)	117.0	44.82	67.04
	CFS	100	64.4 (44.82)	113.0	48.48	80.26	100	63.3 (44.37)	118.5	47.59	79.05
10	FFS	100	77.0 (43.04)	111.1	64.35	89.62	100	53.6 (38.67)	116.3	42.25	64.95
	CFS	100	66.1 (46.00)	113.5	47.15	85.13	100	59.9 (45.16)	111.6	41.29	78.57
11	FFS	100	76.4 (43.38)	116.6	64.95	87.76	100	67.8 (45.08)	116.8	55.98	79.69
	CFS	97.4	67.8 (45.46)	117.4	52.82	82.70	100	53.4 (45.53)	120.0	38.64	68.16
12	FFS	100	94.4 (39.22)	104.7	82.92	105.95	100	69.2 (44.43)	116.7	56.18	82.26
	CFS	96.8	81.5 (44.75)	120.0	64.83	98.24	93.5	77.9 (44.94)	118.5	60.81	95.00
13	Floor	100	86.7 (40.84)	108.0	74.32	99.15	100	67.9 (43.68)	112.0	54.62	81.17
	Foam	100	81.6 (40.49)	108.7	65.24	97.94	100	60.5 (40.04)	118.5	44.31	76.65

Table 3 provides the means, standard deviations (SD), ranges, and upper and lower bounds for the 95% confidence intervals (CIs) of the means for the 4 standing positions (SPs) on a firm floor surface (FFS) and a compliant floor surface (CFS) in one-year age groups. The percentages of children in each age group willing to attempt each SP are also provided.

## Data Availability

Data are unavailable to others due to privacy restrictions enforced by the United States government (HIPAA, FERPA). The protocol for this study approved by Belmont University and Damen University Institutional Review Boards does not include permission for data sharing. The authors are willing to collaborate with others who would find this research valuable.

## Appendix A. Demographics of Study Participants

**Age**	**N**		**Height**	**Weight**	**BMI**	**Floor Surface (N)**	
**Years**	**Boys**	**Girls**	**Mean (SD)**	**Mean (SD)**	**Mean (SD)**	**FFS**	**CFS**
2	30	26	35.3 (2.53)	31.3 (5.18)	17.8 (3.42)	56	34
3	41	39	38.8 (2.52)	36.5 (6.92)	17.1 (2.39)	80	50
4	42	57	41.7 (2.45)	40.9 (6.91)	16.4 (1.91)	99	48
5	40	41	44.6 (2.52)	46.3 (9.04)	16.3 (2.41)	81	45
6	31	42	47.4 (3.02)	52.0 (10.10)	16.1 (2.19)	73	53
7	40	39	49.9 (2.87)	59.4 (10.18)	16.8 (2.47)	79	45
8	45	33	52.3 (3.32)	68.2 (15.8)	17.5 (3.19)	78	53
9	31	34	53.8 (4.00)	75.4 (17.98)	18.2 (3.47)	65	33
10	27	20	56.8 (2.58)	81.6 (18.21)	17.7 (3.02)	47	25
11	36	22	59.1 (4.92)	91.2 (23.33)	18.3 (3.63)	58	39
12	28	19	62.1 (3.79)	111.0 (24.17)	20.1 (3.49)	47	31
13	22	22	63.4 (5.41)	120.5 (32.32)	20.8 (4.03)	44	26
**Total**	**413**	**394**				**807**	**482**
Appendix A provides a conversion of Table 1 from metric units of measure to US customary units of measure to facilitate ease of access. Means (standard deviations) for height given in inches and weight in pounds. BMI was calculated using the following formula: BMI = 703 × weight (pounds)/height (inches) squared. All other aspects of this appendix are consistent with Table 1.							

## Appendix B. Results of Two-Way ANOVA with Sex and Age for Each Standing Position and Floor Surface

**Static Measure**	**Effects**	**F**	**df1/df2**	***p***
Standing Unsupported FFS				
	Sex	1.34	1, 779	<0.001
	Age	73.48	11, 779	<0.001
	Sex × age	1.60	11, 779	=0.095
Standing, Feet Together FFS				
	Sex	1.83	1, 772	=0.176
	Age	88.94	11, 772	<0.001
	Sex × age	1.08	11, 772	=0.375
Tandem FFS				
	Sex	4.78	1, 747	=0.029
	Age	45.36	11, 747	<0.001
	Sex × age	0.79	11, 747	=0.655
Single Limb FFS				
	Sex	1.22	1, 755	=0.029
	Age	44.40	11, 755	<0.001
	Sex × age	1.37	11, 755	=0.655
Standing Unsupported CFS				
	Sex	0.01	1, 456	=0.943
	Age	27.54	11, 456	<0.001
	Sex × age	1.53	11, 456	=0.116
Standing, Feet Together CFS				
	Sex	0.00	1, 444	=0.993
	Age	39.21	11, 444	<0.001
	Sex × age	0.71	11, 444	=0.731
Tandem CFS				
	Sex	2.15	1, 382	=0.143
	Age	15.43	11, 382	<0.001
	Sex × age	1.14	11,382	=0.332
Single Limb CFS				
	Sex	3.87	1, 405	=0.05
	Age	21.64	11, 405	<0.001
	Sex × age	0.66	11, 405	=0.776
Each static standing position (SP) was analyzed separately for each floor surface (FFS, firm floor surface; CFS, compliant floor surface). F statistics for each of the main effects and interactions, degrees of freedom, and *p* (probability value) are provided.				

## Appendix C. Repeated-Measures Two-Way ANOVA Results for Floor and Age Effects on Static Standing Positions

**Static Measure**	**Effects**	**F**	**df1/df2**	***p***
Standing Unsupported				
	Floor	9.10	1, 468	<0.001
	Age	44.55	11, 468	<0.001
	Floor × age	0.41	11, 468	=0.95
Standing, Feet Together				
	Floor	12.34	1, 458	<0.001
	Age	57.19	11, 458	<0.001
	Floor × age	1.10	11, 458	=0.36
Tandem				
	Floor	66.16	1, 395	<0.001
	Age	28.28	11, 395	<0.001
	Floor × age	1.74	11, 395	<0.063
Single Limb				
	Floor	45.63	1, 417	<0.001
	Age	33.57	11, 417	<0.001
	Floor × age	2.42	11, 417	<0.006
Each static measure was analyzed separately. F statistics for each of the main effects and interaction, degrees of freedom, and *p* (probability value) are provided.				

## Appendix D. Repeated-Measures Two-Way ANOVA Results for Altered Visual Input and Age Effects on Static Standing Positions for Each Floor Surface

**Static Measure**	**Effects**	**F**	**df1/df2**	***p***
Standing Unsupported FFS				
	Eyes	23.21	1, 754	<0.001
	Age	88.88	11, 754	<0.001
	Eyes × age	2.16	11, 754	=0.015
Standing Unsupported CFS				
	Eyes	25.79	1, 425	<0.001
	Age	32.55	11, 425	<0.001
	Eyes × age	3.67	11, 425	<0.001
Standing unsupported was analyzed separately for each floor surface (firm floor surface [FFS] and compliant floor surface [CFS]). F statistics for each of the main effects and interaction, degrees of freedom and *p* (probability value) are provided. “Eyes” indicates eyes open vs. eyes closed				

## Appendix E. Percentage of Children in Time Quartiles for SU with Eyes Closed by Age Group for Each Floor Surface

	**Standing Unsupported Time (Eyes Open)**						**Standing Unsupported Time (Eyes Closed)**				
**Age**	**Surface**	**% Able to do Task**	**Mean (SD)**	**Range**	**95% CI**		**% Able to do Task**	**Mean (SD)**	**Range**	**95% CI**	
					**Lower**	**Upper**				**Lower**	**Upper**
					**Bound**					**Bound**	
2	FFS	98.2	37.1 (38.44)	117.9	26.68	47.46	53.6	33.0 (23.17)	69.8	19.62	46.37
	CFS	100	35.7 (34.39)	119.0	23.73	47.73	25	26.8 (29.43)	116.4	9.80	43.78
3	FFS	100	50.7 (43.24)	117.4	41.05	60.30	86.3	53.4 (38.81)	116.0	39.85	66.93
	CFS	100	59.5 (46.16)	118.5	46.38	72.62	42.5	38.7 (40.59)	118	24.57	52.90
4	FFS	100	71.0 (41.91)	117.4	62.66	79.38	98	75.7 (42.08)	118.8	62.94	88.52
	CFS	100	73.4 (44.94)	115.0	60.36	86.46	44.4	59.2 (43.49)	116.0	45.99	72.44
5	FFS	100	99.0 (34.76)	104.5	91.30	106.67	100	92.6 (5.82)	106.3	80.82	104.34
	CFS	100	94.5 (38.72)	113.5	82.85	106.12	51.9	74.6 (45.96)	118.4	60.32	88.96
6	FFS	98.6	111.3 (25.49)	114.0	105.33	117.31	100	109.8	26.5	102.52	117.11
	CFS	100	105.8 (31.20)	104.8	97.20	114.40	72.6	90.3 (41.36)	116.9	78.88	101.69
7	FFS	100	109.5 (26.29)	98.7	103.64	115.41	100	110.3	24.6	102.92	117.70
	CFS	100	107.5 (24.64)	98.8	100.09	114.90	57	107.8 (27.12)	109.4	99.66	115.96
8	FFS	100	120.0 (0.00)	0.0	120.00	120.00	98.7	120 (0)	0.0	120.00	120.00
	CFS	100	114.2 (23.61)	119.0	107.70	120.71	65.4	116.5 (15.97)	97.8	112.04	121.02
9	FFS	100	118.4 (7.13)	45.0	116.66	120.19	100	116.7 (2.16)	61.2	112.30	121.11
	CFS	100	115.9 (20.19)	115.0	108.78	123.10	50.8	108.4 (30.03)	113.0	97.74	119.04
10	FFS	100	119.5 (3.21)	22.0	118.59	120.47	100	120 (0)	0.0	120.00	120.00
	CFS	100	116.3 (18.66)	93.3	108.57	123.97	53.2	114.6 (20.82)	99.2	106.00	123.19
11	FFS	100	117.7 (11.39)	67.5	114.70	120.69	100	115.5 (19.17)	106.7	109.27	121.69
	CFS	100	110.6 (25.90)	95.0	102.16	118.95	67.2	107.1 (30.77)	111.6	97.07	117.02
12	FFS	100	117.0 (14.41)	75.3	112.77	121.23	100	120 (0)	0.0	120.00	120.00
	CFS	100	119.9 (0.34)	1.9	119.81	120.06	66	119.3 (4.13)	23.0	117.74	120.77
13	FFS	100	120.0 (0.00)	0.0	120.00	120.00	100	120 (0)	0.0	120.00	120.00
	CFS	100	120.0 (0.00)	0.0	120.00	120.00	59.1	119.0 (3.77)	16.8	117.44	120.49
This table provides the means, standard deviations (SD), ranges, and upper and lower bounds for the 95% confidence interval (CI) of the means for the standing unsupported (SU) on firm floor surface (FFS) and compliant floor surface (CFS) in one-year age groups with eyes open and eyes closed. The percentages of children in each age group willing to attempt each task are also provided.											

## Appendix F. Percentage of Children in Time Quartiles for Standing Unsupported on Firm and Compliant Floor Surfaces by Age Group with Eyes Closed

These figures depict the percentages of children in time quartiles by floor surface: (**a**) Firm Floor Surface (FFS) and (**b**) Compliant Floor Surface (CFS) and age groups. Quartiles represent intervals of standing times in seconds (0–29, 30–59, 60–89, 90–119) and those attaining the maximum time of 120 seconds, as these children could potentially have maintained the position longer.

## Appendix G. Results for Multiple Linear Regression for Each Standing Position and Floor Surface

**Static Measure**	**R^2^**	**Model Statistics**	**Constant**	**Age**	**Height**	**BMI**
Standing Unsupported FFS	0.367	F_3,787_ = 153.83 *p* < 0.001	30.842 *t* = 2.472 *p* = 0.014	0.415 *t* = 5.475 *p* < 0.001	0.223 *t* = 2.979 *p* = 0.003	−0.079 *t* = −2.657 *p* = 0.008
Standing Feet Together FFS	0.437	F_3,780_ = 203.223 *p* < 0.001	7.472 *t* = 0.582 *p* = 0.561	0.445 *t =* 6.243 *p* < 0.001	0.247 *t* = 3.512 *p* < 0.001	−0.069 *t* = −2.420 *p* = 0.016
Tandem FFS	0.388	F_2,757_ = 241.401 *p* < 0.001	−57.795 *t* = −4.486 *p* < 0.001	0.391 *t* = 5.314 *p* < 0.001	0.245 *t* = 3.333 *p* < 0.001	NA
Single Limb FFS	0.388	F_2,764_ = 235.815 *p* < 0.001	−6.657 *t* = −1.061 *p* = 0.289	0.636 *t* = 21.301 *p* < 0.001	NA	−0.072 *t* = −2.412 *p* = 0.016
Standing Unsupported CFS	0.294	F_2,473_ = 100.077 *p* < 0.001	19.855 *t* = 1.432 *p* = 0.153	0.335 *t* = 3.436 *p* < 0.001	0.221 *t* = 2.273 *p* < 0.023	NA
Standing Feet Together CFS	0.359	F_3,462_ = 87.861 *p* < 0.001	16.286 *t* = 1.003 *p* = 0.316	0.396 *t* = 4.237 *p* < 0.001	0.239 *t* = 2.576 *p* = 0.01	−0.075 *t* = −1.925 *p* = 0.055
Tandem CFS	0.294	F_1,403_ = 56.142 *p* < 0.001	−15.913 *t* = −3.051 *p* = 0.002	0.544 *t* = 13.008 *p* < 0.001	NA	NA
Single Limb CFS	0.334	F_2,424_ = 107.936 *p* < 0.001	3.342 *t* = 0.230 *p* = 0.818	0.727 *t* = 7.754 *p* < 0.001	−0.166 *t* = 1.768 *p* = 0.078	NA
Backward linear regression was completed for each standing position and floor surface (FFS, firm floor surface; CFS, compliant floor surface). Weight was removed from consideration due to collinearity with height. Adjusted R^2^ indicates the proportion of variation explained by the model. Model statistics provide the F statistic, degrees of freedom (subscripts) and *p* value. Constant is the intercept for the model along with *t* statistic and *p* value. For each independent variable, the standardized coefficient with *t* statistic and *p* value are provided. NA—variable was not significant and dropped from the model.						
